# Lung adenocarcinoma presenting as a solitary gingival metastasis: a case report

**DOI:** 10.1186/1752-1947-5-202

**Published:** 2011-05-25

**Authors:** Armando Orlandi, Michele Basso, Mariantonietta Di Salvatore, Francesco Federico, Alessandra Cassano, Carlo Barone

**Affiliations:** 1 Division of Medical Oncology, Catholic University of Sacred Heart, Rome, Italy; 2 Department of Pathology, Catholic University of Sacred Heart, Rome, Italy

## Abstract

**Introduction:**

Gingival metastases are very rare and generally occur in disseminated tumors. We report a case of solitary gingival metastasis of lung cancer.

**Case presentation:**

We report the case of a 74-year-old asymptomatic Caucasian woman affected by a rapidly growing, painless gingival swelling. Histopathologic examination of the excisional biopsy showed metastasis of poorly differentiated thyroid transcription factor 1-positive adenocarcinoma. A total-body computed tomographic scan revealed a tumor of the right lung lower lobe with ipsilateral, mediastinal lymph node swelling. Moreover, bone scintigraphy revealed no bone metastases. No other metastases were found, so we planned a multi-modal therapeutic approach with a curative intent. However, the tumor proved to be intrinsically resistant and highly aggressive.

**Conclusion:**

The presentation of solitary gingival metastasis is exceptional. In view of its rapid clinical evolution, our case confirms that gingival metastasis is an important prognostic factor. This behavior raises the question whether the poor prognosis for patients with tumors with oral metastases depends on its diffuse spread or on its highly malignant nature.

## Introduction

Oral metastatic tumors are rare, comprising approximately 1% of all oral tumors [1]. The jawbones are affected in 90% of the cases, whereas metastases to the soft tissues of the oral cavity occur very rarely and mostly involve the gingiva (54% of soft tissue metastases), followed by the alveolar mucosa or the tongue [2,3]. Metastases may reach the oral cavity hematogenously, mainly through inversion of the venous flow in the cervical Batson's plexus [4]. Alternatively, exfoliating cancer cells might be implanted in the oral mucosa by retrograde spreading along the respiratory tract or by cough [5]. The hyper-vascularization in inflamed periodontal tissues may be a causative factor [6]. In 30% of cases, oral metastasis is the first manifestation of cancer, but it is often a sign of advanced disease with multi-metastatic involvement [7]. In fact, survival after recognition of gingival metastasis ranges from a few weeks to less than six months, with five-year survival lower than 5% [7-10]. The poor prognosis related to this condition points out the importance of differentiating oral metastases from benign lesions, which often is achievable only by surgical excision. The case that we report here shows that a gingival metastasis may be the only presenting sign of lung adenocarcinoma, but it remains associated with a dismal outcome.

## Case presentation

**Figure 1:**
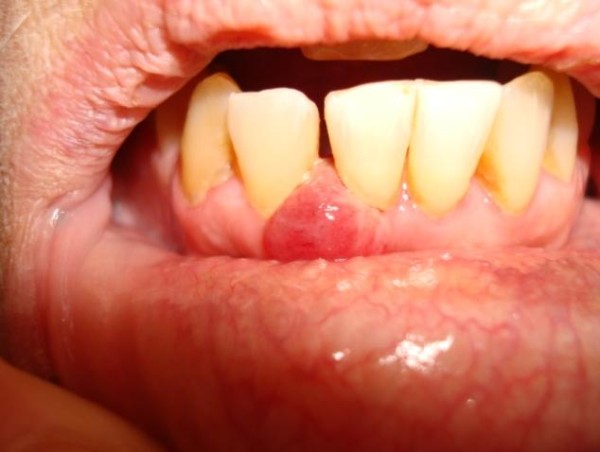
**Intra-oral view of the lesion developing in front of right jaw incisors**.

**Figure 2:**
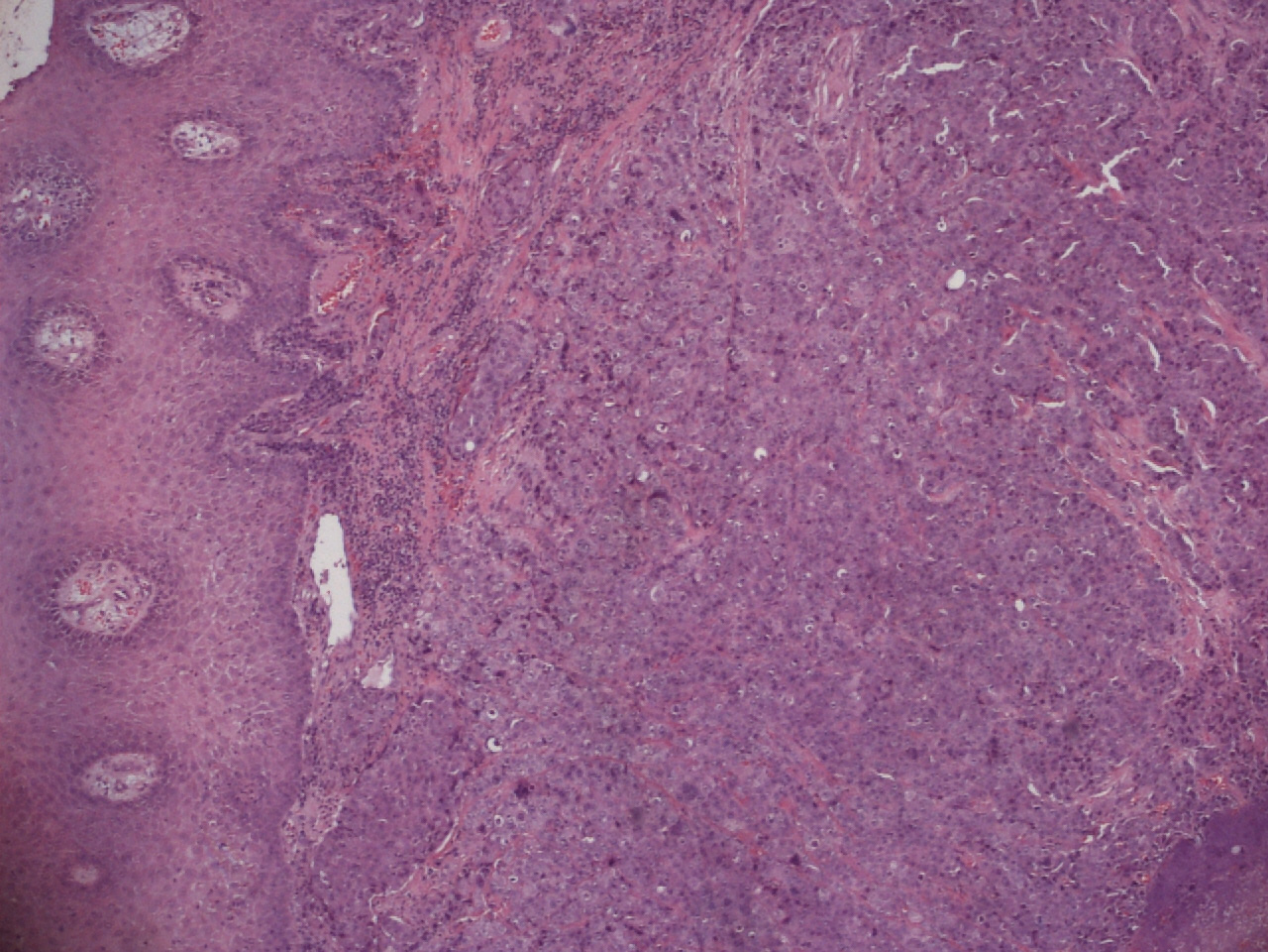
**Histopathologic study showing proliferation of adenocarcinoma cells below the gingival epithelium (hematoxylin and eosin stain; original magnification, × 4)**.

**Figure 3:**
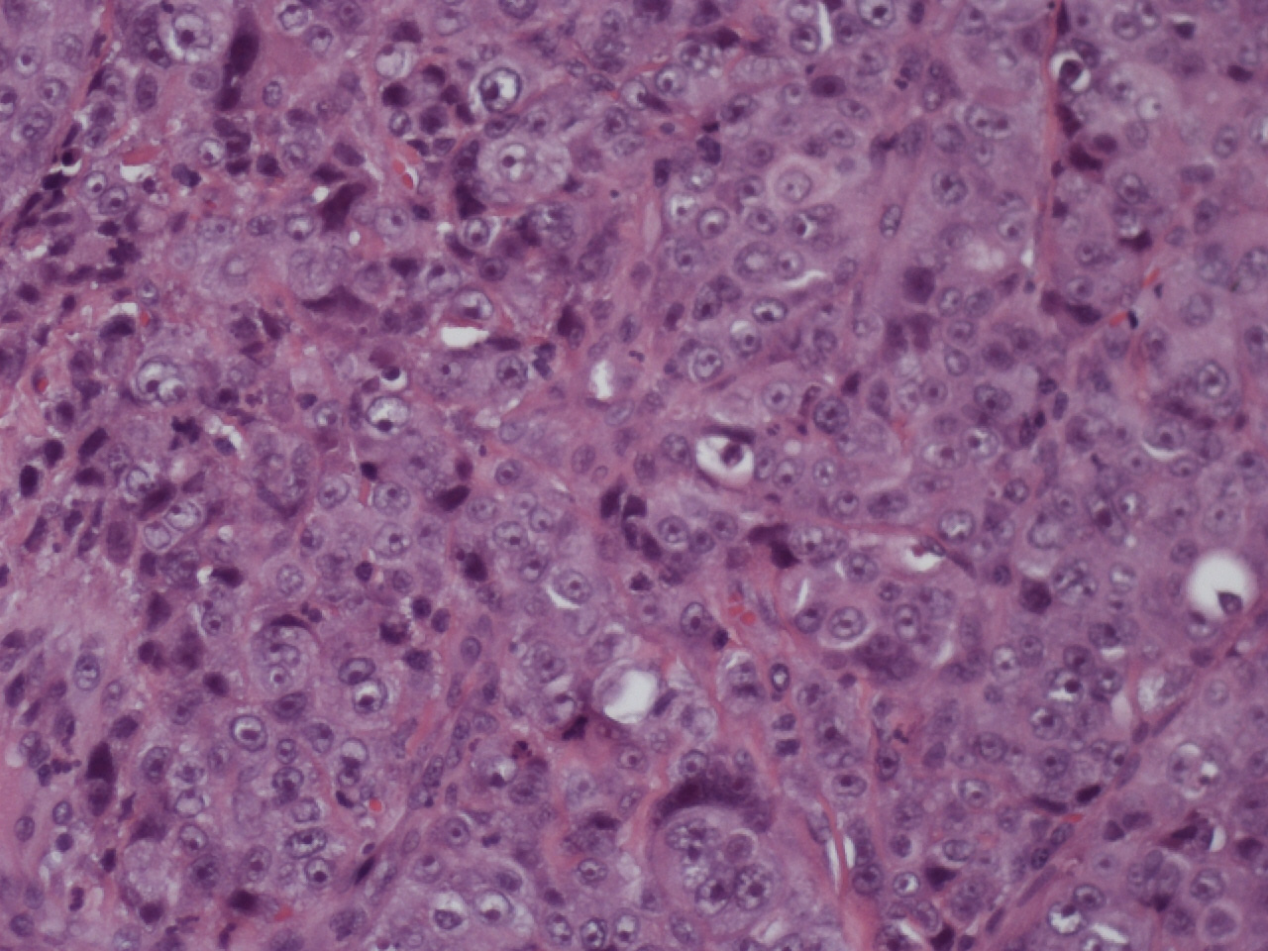
**Histopathologic study showing proliferation of adenocarcinoma cells below the gingival epithelium (hematoxylin and eosin stain; original magnification, × 20)**.

**Figure 4:**
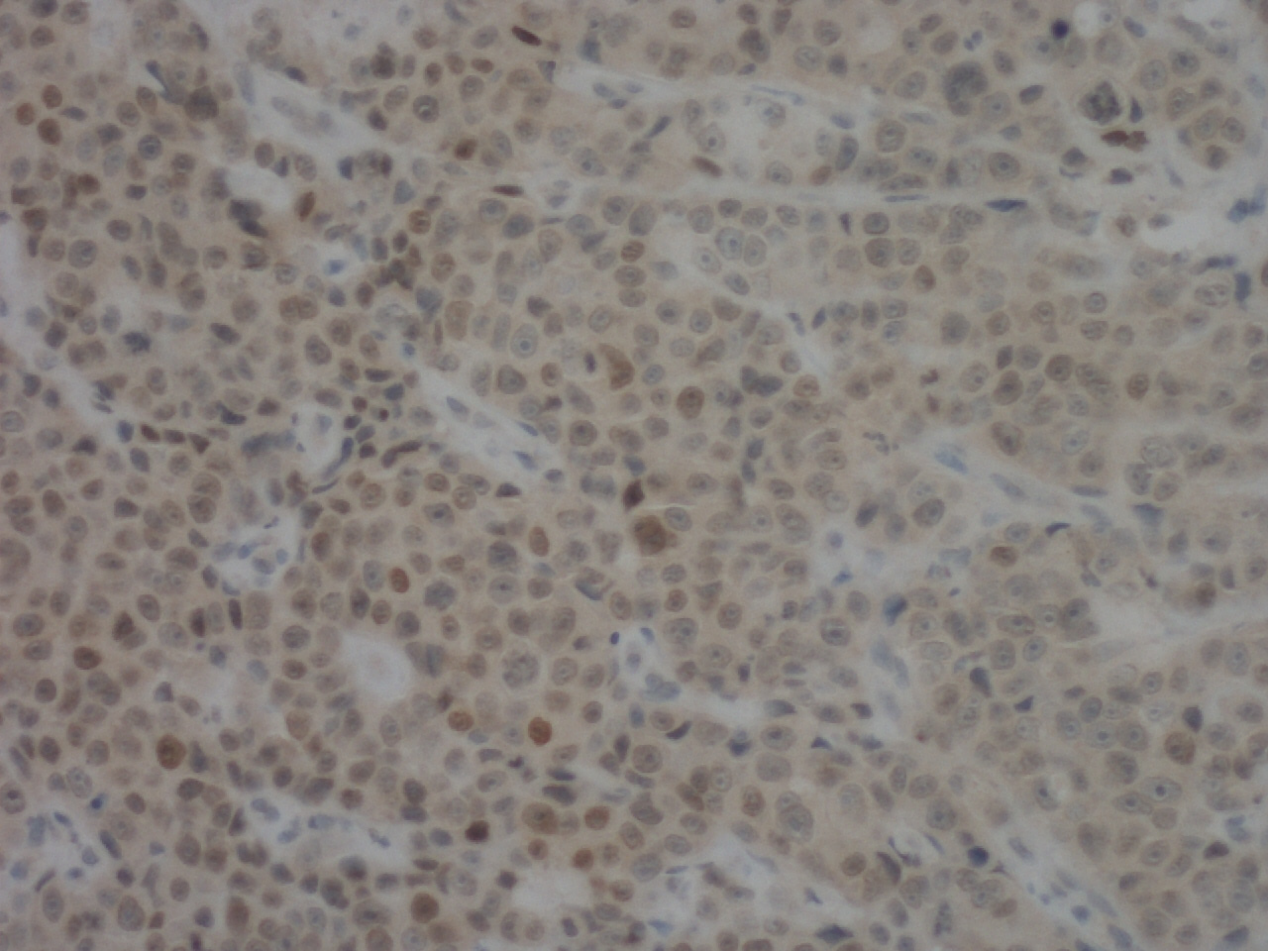
**The tumor cells were immunoreactive for thyroid transcription factor 1 (original magnification, × 20)**.

**Figure 5:**
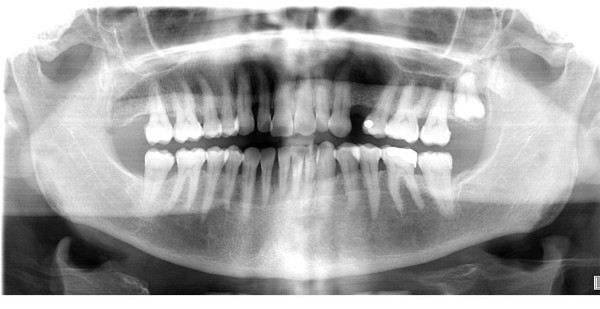
**Panoramic radiography showing generalized alveolysis**.

An apparently healthy, 74-year-old Caucasian woman who was a non-smoker and had no history of alcohol addiction presented with swelling of the vestibular gingival mucosa at the level of the lower right incisors (Figure 1). No other pathologic finding was noticed during the physical examination. She underwent an excisional biopsy of the lesion, and histopathologic immunohistochemistry showed a poorly differentiated adenocarcinoma expressing cytokeratin 7 and thyroid transcription factor 1, whereas cytokeratins 5, 6 and 20 were absent. The pattern suggested a metastasis of lung cancer (Figures 2,3,4). The total-body computed tomographic (CT) scan with contrast-enhancing medium revealed a 7.4 cm-sized tumor of the lower lobe of the right lung with metastases to the ipsilateral mediastinal lymph nodes (cT3N2). No other metastases were detected, and her bone scan was also negative. An orthopantomogram of the dental arches excluded metastases to the jawbones (Figure 5). After multi-disciplinary clinical evaluation, sequential treatment was planned, including neoadjuvant chemotherapy (ChT) followed by concomitant chemoradiation and surgery. Platinum-based combination therapy was selected, but cisplatinum was excluded because the patient had low-grade renal insufficiency with a serum creatinine level of 1.8 mg/dL. Therefore, carboplatin area under the curve 6 on day one and gemcitabine (1000 mg/mq on days one and eight) every three weeks were started. Two months later, after she had undergone three cycles of ChT, her CT scan showed clear expansion of the primary tumor with diffuse infiltration of the right lung. A second-line treatment with docetaxel was attempted, but the tumor rapidly progressed and the patient died six weeks later as a result of respiratory failure.

## Discussion

Metastatic tumors to the oral region are rare and mostly produced by breast, lung and kidney cancer, but other tumors may be also included [6]. Bone involvement is much more frequent than soft tissue involvement, and in the latter case lung cancer is the most common primary source. Hirshberg *et al*. [6] reviewed cases of oral metastases reported from 1916 to 1991 and found 157 cases of oral soft tissue metastases, 86 of which had gingival localization. The primary tumors were located in the lung (25.5%), kidney (15.1%), bone (10.4%), breast (9.3%) and liver (8.1%). Yoshii *et al*. [11] estimated that the probability of lung cancer involving a diagnosis of gingival metastasis is about 10% to 20%. Other authors have emphasized that the prognosis of patients with oral metastases is very poor, with a median survival of less than six months, mainly because of the fact that oral metastases are an expression of a multi-metastatic disease [12]. A recent review of 39 patients with oral metastases confirmed a median survival of 5.2 months without significant differences according to oral localization or to the site of the primary cancer [13]. In our patient, oral localization was the only metastasis detectable at presentation. To the best of our knowledge, no other similar cases have been described in the literature, and this calls attention to the importance of recognition of metastases to oral soft tissues. Most gingival lesions in patients with prior or current non-oral malignancies are not metastases [14]. Generally, gingival or oral mucosal metastases extend from mandibular or maxillary lesions and spread beyond the peri-osteum to cause visible gingival or oral mucosal masses [14]. Therefore, gingival metastases are polypoid or exophytic and highly vascularized, and bleeding is very common [8-10,15-17]. The same characteristics are also displayed by a number of benign lesions, such as pyogenic granuloma (or vascular epulis), peripheral giant cell granuloma (giant cell epulis) or fibrous epulis [18]. From a clinical point of view, the aspects suggestive of malignancy are only the rapid growth and the propensity for either necrosis or hemorrhage. In these cases, the possibility of metastasis should be kept in mind, and biopsy is mandatory.

In our patient, no other metastases were found; therefore, we planned a multi-modal therapeutic approach with a curative intent. However, the tumor proved intrinsically resistant and highly aggressive. This behavior raises the question whether the poor prognosis of patients with tumors with oral metastases depends on their diffuse spread or on their highly malignant nature. Early detection might be important in metastases from chemosensitive tumors, whereas chemoresistant tumors, such as lung cancer, the present therapeutic strategies are largely ineffective, and oral metastases should be considered as only a negative prognostic factor.

## Conclusion

In view of the rapid clinical evolution, in spite of the fact that this is a single case report and no clear diagnostic recommendations can be made on the basis of a single report, the present case of our patient supports the fact that gingival metastasis is an important prognostic factor. Thus, given the malignant potential and the diagnostic value of a gingival metastasis, it is essential to carry out the excision of any presumed benign tumor within healthy boundaries and to ask for a systematic histopathological examination.

## Consent

Written informed consent was obtained from the patient for publication of this case report and any accompanying images. A copy of the written consent is available for review by the Editor-in-Chief of this journal.

## Competing interests

The authors declare that they have no competing interests.

## Authors' contributions

OA collected the data and was involved in drafting the manuscript. DM and FF participated in the acquisition of data. BM, CA and BC were involved in drafting the manuscript or revising it for important intellectual content. All authors read and approved the final manuscript.
